# Novel Targets, Novel Treatments: The Changing Landscape of Non-Small Cell Lung Cancer

**DOI:** 10.3390/cancers15102855

**Published:** 2023-05-21

**Authors:** Dorine de Jong, Jeeban P. Das, Hong Ma, Jacienta Pailey Valiplackal, Conor Prendergast, Tina Roa, Brian Braumuller, Aileen Deng, Laurent Dercle, Randy Yeh, Mary M. Salvatore, Kathleen M. Capaccione

**Affiliations:** 1 Center for Cell Engineering, Memorial Sloan Kettering Cancer Center, 1275 York Avenue, New York, NY 10065, USA; ddejong990@gmail.com; 2 Department of Radiology, Memorial Sloan Kettering Cancer Center, 1275 York Avenue, New York, NY 10065, USA; dasj@mskcc.org (J.P.D.); ray9006@med.cornell.edu (R.Y.); 3 Department of Radiology, Columbia University Irving Medical Center, New York, NY 10032, USA; hym2103@cumc.columbia.edu (H.M.); jp3828@cumc.columbia.edu (J.P.V.); cprender@student.touro.edu (C.P.); tir7004@nyp.org (T.R.); braumuller.b@northeastern.edu (B.B.); ld2752@cumc.columbia.edu (L.D.); ms5680@cumc.columbia.edu (M.M.S.); 4 Department of Hematology and Oncology, Novant Health, 170 Medical Park Road, Mooresville, NC 28117, USA; a.deng@novanthealth.org

**Keywords:** lung cancer, targeted therapies, molecular diagnostics, PET imaging

## Abstract

**Simple Summary:**

Non-small cell lung cancer treatment has undergone a revolution in the past decade owing to the discovery of mutations that drive carcinogenesis and the development of molecular testing and treatments that act on them. Here, we detail the way in which lung cancer is currently diagnosed and treated in light of these developments. We focus on some of the key mutations for which targeted therapies have been developed and the trials that led to their approval. We hope to provide a comprehensive review of the current diagnosis and management of non-small cell lung cancer.

**Abstract:**

Treatment of non-small cell lung cancer (NSCLC) has undergone a paradigm shift. Once a disease with limited potential therapies, treatment options for patients have exploded with the availability of molecular testing to direct management and targeted therapies to treat tumors with specific driver mutations. New in vitro diagnostics allow for the early and non-invasive detection of disease, and emerging in vivo imaging techniques allow for better detection and monitoring. The development of checkpoint inhibitor immunotherapy has arguably been the biggest advance in lung cancer treatment, given that the vast majority of NSCLC tumors can be treated with these therapies. Specific targeted therapies, including those against KRAS, EGFR, RTK, and others have also improved the outcomes for those individuals bearing an actionable mutation. New and emerging therapies, such as bispecific antibodies, CAR T cell therapy, and molecular targeted radiotherapy, offer promise to patients for whom none of the existing therapies have proved effective. In this review, we provide the most up-to-date survey to our knowledge regarding emerging diagnostic and therapeutic strategies for lung cancer to provide clinicians with a comprehensive reference of the options for treatment available now and those which are soon to come.

## 1. Lung Cancer

**Figure 1:**
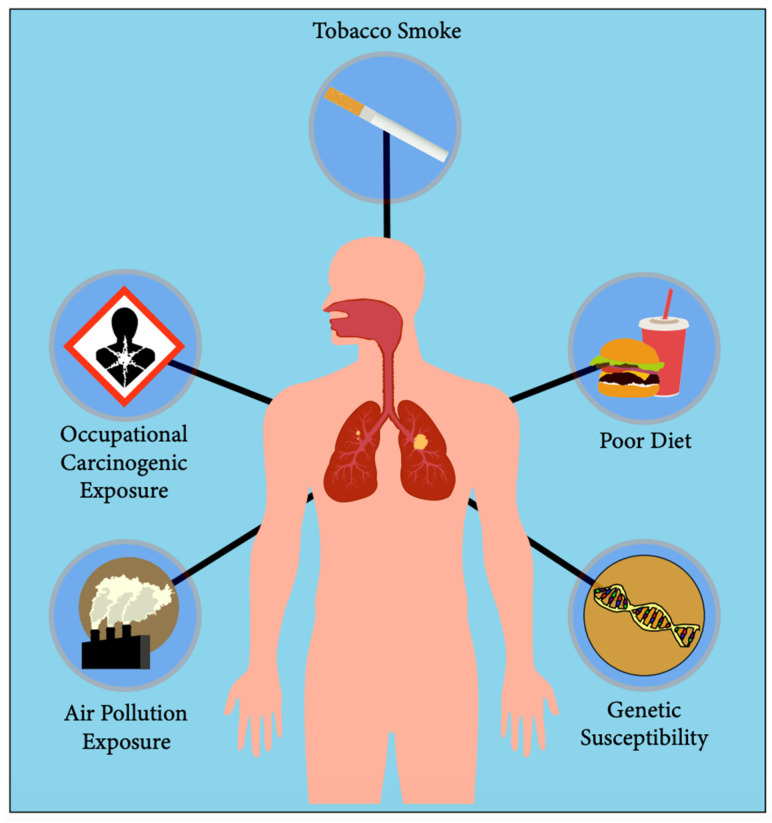
Demonstrates the leading risk factors for lung cancer in the United States.

Lung cancer is the leading cause of cancer death. The two major types are small cell and non-small cell; 85% are non-small cell lung cancer (NSCLC). Within NSCLC, there are three subtypes: adenocarcinoma, squamous cell carcinoma, and large cell carcinoma [1]. Cigarette smoking is the leading cause of lung cancer; however, there are several other risk factors, such as sustained exposure to air pollution, occupational carcinogenic exposures, genetic susceptibility, and poor diet [2] (Figure 1). Many patients with no smoking history or a history of other risk factor exposure also develop lung cancer and there is an urgent need for effective therapies to treat and cure lung cancers. 

Traditionally, NSCLC was treated with surgery, chemotherapy, and radiation therapy alone or in combination depending on the stage of disease. For stages I and II, surgical resection remains the preferred approach for treatment [3]. 

Targeted therapies offer a more precise approach of killing cancer cells than chemotherapy. In NSCLC, several key driver mutations have been identified [1]. Therapies against these mutations have demonstrated excellent anti-cancer efficacy [4]. Table 1 summarized some of the key actionable mutations in NSCLC. Several key NSCLC mutations, however, are yet to be successfully targeted and require further study. Increasing the number of mutations that can be successfully targeted will allow for a more precise approach to lung cancer treatment. The treatment for an NSCLC patient should include a tailored regimen including imaging, molecular testing, and targeted therapy alongside traditional approaches such as chemotherapy, radiation therapy, and surgical resection. 

**Table 1:** Summarizes the most common genetic mutations in lung cancer and how they are commonly screened for in current clinical practice.

Genes	Common Mutations	Mutation Prevalence	Screening Protocol
*EGFR* [5,6]	Exon 19 deletion * Exon 20 T790M Exon 21 L858R * (*: Present in 40% of patients)	Adenocarcinoma: 38% Non-adenocarcinoma: 12% Western: 10–15% Asia: 30–40% African-American: 20% Male: 24% Female: 44%	Direct DNA sequencing is the gold standard if sample is more than 50% tumor content. If not, PCR preferred.
*MET* [7,8,9,10]	*TPR-MET* fusion Exon 14 skip *c-MET*-N375S	Adenosquamous carcinoma: 5% Adenocarcinoma: 3% Squamous cell carcinoma: 2% Western: 12% Asian: 1–4% African-American: 10%	FISH assay is the gold standard; next-generation sequencing is reliable only for a high-level of *MET* gene amplification.
*ALK* [11,12]	Fusion with: -*TMP3-TFG-CLTCL1* -*ATIC*-*EML4* * (*: Most common, ~30% of all *ALK* fusions)	Adenocarcinoma: 5% Western: 5% Asian: 5%	FISH assay is the gold stand; RT-PCR is also FDA-approved for only *EML4-ALK* fusion.
*RET* [13]	*RET-KIF5B* fusion * RET-CCDC6 fusion *RET-NCOA4* fusion (*: Detected in EGFR inhibitor resistant cancers)	All NSCLC: 1.5% Adenocarcinoma: 1.7% Age > 60: 2.0% Age < 60: 1.0% Male: 0.9% Female: 1.7%	Next-generation sequencing and FISH assays are comparable; however, FISH demonstrated lower sensitivity for *RET-NCOA4 fusions*
*HER2* [14]	Exon 20 12 bp insertion Exon 20 L755S Exon 20 G776C	Adenocarcinoma: 2–4%	Mutation: next-generation sequencing, Amplification: FISH, Overexpression: IHC.

## 2. Imaging Lung Cancer

**Figure 2:**
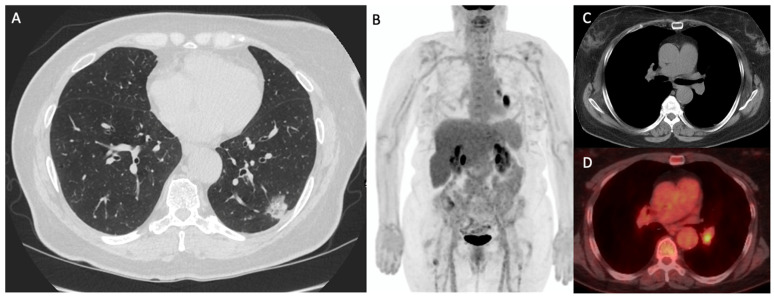
Demonstrates a 62-year-old woman with biopsy-proven left lung adenocarcinoma. (**A**) Axial CT showing part-solid pulmonary nodule in the left lower lobe, with biopsy demonstrating adenocarcinoma (Stage: T1N1M0). (**B**) MIP, (**C**) CT and (**D**) fused ^18^F FDG-PET/CT maximum intensity projection showing radiotracer avid lung malignancy and uptake in a left hilar node, indeterminate on prior CT, and confirmed as nodal metastasis on subsequent biopsy.

**Figure 3:**
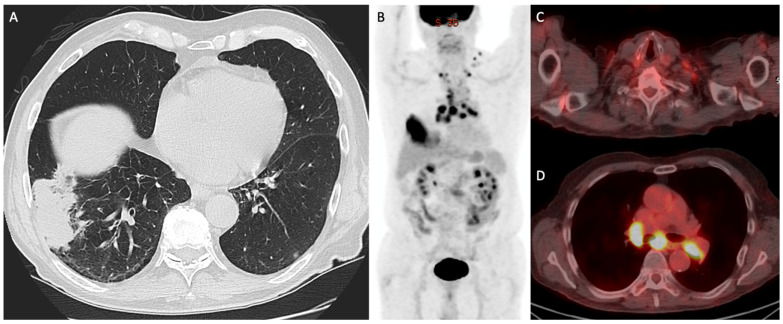
Demonstrates a 68-year-old man with biopsy-proven right lung adenocarcinoma (Stage: T3N3M0). (**A**) Axial CT showing a mass in the lateral right lower lobe, abutting adjacent pleura, with biopsy demonstrating adenocarcinoma. (**B**) ^18^F-FDG-PET/CT maximum-intensity projection and (**C**,**D**) fused images showing multiple enlarged, avid ipsilateral and contralateral mediastinal and supraclavicular lymph nodes, suspicious for metastases, with the left supraclavicular node subsequently biopsied confirming malignancy.

**Figure 4:**
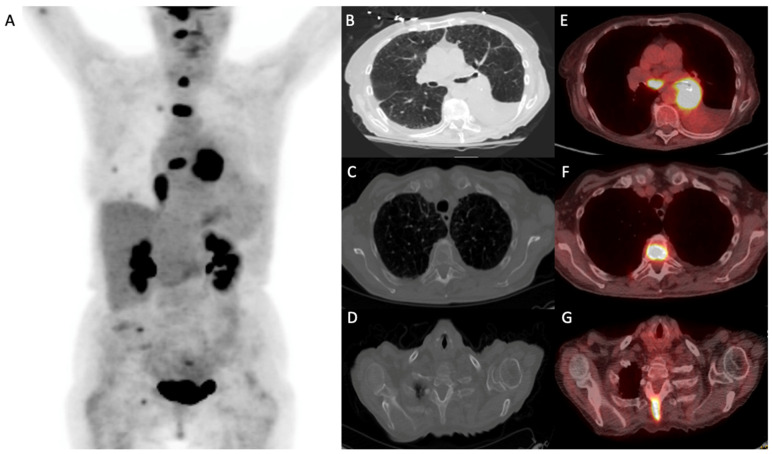
Demonstrates a 68-year-old man with biopsy-proven left lung squamous cell carcinoma (Stage: T4N2M1c). (**A**) ^18^F-FDG-PET/CT maximum intensity projection showing left lower lobe perihilar neoplasm invading the mediastinum, narrowing the left main bronchus with post-obstructive atelectasis; a biopsy demonstrated squamous cell carcinoma. (**B**–**D**) CT images and (**E**–**G**) fused images demonstrate enlarged FDG-avid subcarinal node suspicious for metastasis and multiple FDG-avid osseous lesions, with some lytic, and others CT-occult, consistent with metastases.

Imaging algorithms for the evaluation of non-small cell lung cancer (NSCLC) have evolved over the past decades. While chest radiography and computed tomography (CT) remain important tools in screening and anatomic localization, the addition of metabolic assessment with positron emission tomography (PET)/CT has been shown to impact prognosis and to help guide a patient’s treatment strategies [15,16] (Figure 2, Figure 3 and Figure 4). 

The most critical criteria that impact prognosis on imaging include the tumor size (T), local mediastinal and hilar nodal metastases (N) and the presence of distant metastases (M) [17]. 

The most commonly used and rigorously studied radiotracer in PET/CT cancer imaging is 18-fluorine (^18^F)-fluorodeoxyglucose (FDG), and its use has become the standard of care for the initial staging, prognostication, and evaluation of treatment response in patients with NSCLC [15,18,19,20,21,22].

While ^18^F-FDG is an established radiotracer for detecting and staging lung cancer, it lacks specificity [23]. Therefore, novel radiopharmaceuticals have been developed and evaluated for the purpose of therapeutic risk stratification and monitoring of the treatment response in NSCLC patients. These agents include ^18^F-fluorothymidine (^18^F-FLT), ^18^F-fluoromisonidazole (^18^F-FMISO), targeted integrin imaging, and more recently, radiolabeled fibroblast activation protein inhibitors (FAPI) [23,24,25]. ^18^F-FLT is a marker of cellular proliferation and is highly correlated to Ki67 expression in NSCLC [26,27,28]. Tian et al. imaged pulmonary nodules in 55 patients with both ^18^F-FLT and ^18^F-FDG and found that the combined use of both radiotracers improved the nodule characterization compared to the individual tracers, with sensitivity and specificity of 100% and 90%, respectively [29]. ^18^F-FMISO can be used to image and quantify tumor hypoxia [30]. ^18^F-FMISO diffuses passively into tumor cells, is metabolized, and its reduction products bind to intracellular macromolecules and are trapped intracellularly under hypoxic conditions. 

The use of synchronous or sequential multi-tracer PET/CT imaging both pre- and intra-treatment with chemotherapy or radiation therapy could potentially allow for a more personalized NSCLC treatment [31,32]. Huang et al. compared the intratumoral accumulation of ^18^F-FDG, ^18^F-FLT, and ^18^F-FMISO and correlated them to the specific components of the tumor microenvironment in mouse models of human NSCLC [31]. They found that ^18^F-FDG and ^18^F-FMISO displayed similar intratumoral distribution patterns, and both mutually excluded ^18^F-FLT [31]. 

Metabolic imaging of angiogenesis in NSCLC can be achieved by imaging integrins, a group of cell adhesion molecules which are upregulated on activated endothelial cells in association with tumor angiogenesis. RGD peptides can be labeled with ^18^F, ^68^Ga or ^64^Cu for PET imaging. ^18^F-Galacto-RGD PET uptake has been shown to correlate with the immunohistological staining of αvβ3 integrin and ^18^F-AL-NOTA-PRGD2, denoted as ^18^F-alfatide, has also been developed as a radiotracer for targeting integrin αvβ3 [33,34].

**Figure 5:**
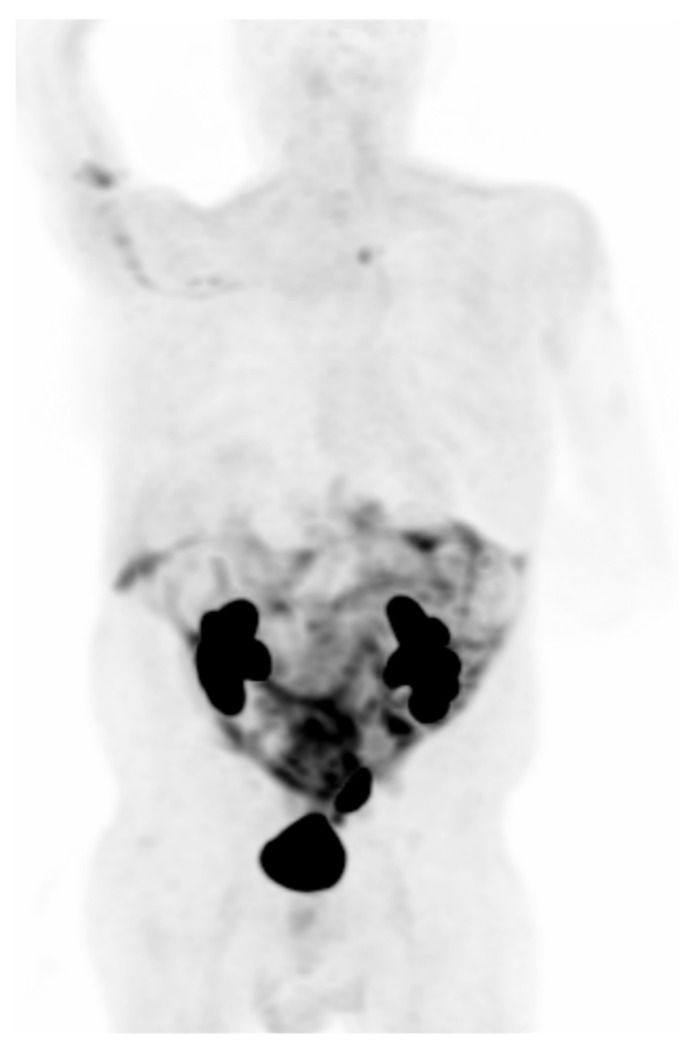
Is a representative ^68^Ga-FAPI maximum intensity projection, negative for malignancy, showing the physiologic uptake of this tracer.

Fibroblast activation protein (FAP) activation and inhibition plays a pivotal role in cancer and inflammation. FAP is a serine protease that is overexpressed in cancer-associated fibroblasts (CAFs) [35]. ^68^Ga-fibroblast activation protein inhibitor (FAPI)-based agents have shown promise as an imaging tool in many types of tumors, including NSCLC [35,36] (Figure 5). 

## 3. Molecular Testing for Lung Cancer

Numerous attempts to detect lung cancer at earlier, more curable stages, have been made. Initial attempts to screen with a chest CT gave equivocal results [37]; however, in 2015, the National Lung Screening Trial Research Team published the results of the largest study of low-dose CT (LDCT), with over 53,000 participants, demonstrating that tri-annual LDCT screening reduced mortality by 20% in comparison to plain film chest radiographs [38]. 

Despite this success, CT imaging provides a limited insight into prognosis and no information on the molecular profile of a tumor, which is essential for administering targeted therapy. Due to tumor heterogeneity, ideal molecular tests would involve whole body molecular imaging or peripheral blood samples using next-generation DNA sequencing or DNA probes, known as “liquid biopsies”.

### 3.1. Next-Generation Sequencing (NGS)

NGS “liquid biopsies” screen for circulating tumor DNA (ctDNA), which are fragments of DNA released by cancer cells into the blood. All tumor sites release ctDNA into the blood, and ctDNA assays can detect cancers with better sensitivity and specificity than biopsy [39]. For lung cancer, ctDNA can screen for KRAS and TP53 mutations two years before diagnosis, facilitating the early use of targeted therapies [40]. A limitation of the use of ctDNA is that DNA fragments are also released by normal hematopoiesis, which may result in false positive results [41]. 

### 3.2. Polymerase Chain Reaction (PCR)

An alternate way to sample ctDNA is using PCR [42]. PCR-based mass spectrometry tests have also been approved in Europe and the USA for genotyping and the detection of mutations in *EGFR*, *KRAS*, and *BRAF*. PCR amplifies samples and then mass-spectrometry identities amplified alleles [43]. RT-PCR has been shown to be 100% sensitive for the detection of *ALK* fusion variants, even when FISH assays were negative [44].

### 3.3. Fluorescence In Situ Hybridization (FISH) Assay

FISH is a technique using molecular probes against a specific DNA sequence, and is considered the gold standard for detecting *ALK* rearrangement, *EML4-ALK*. Although each assay can only detect a single molecular alteration, it has been widely used in the USA since its release in 2011 [44,45]. 

**Figure 6:**
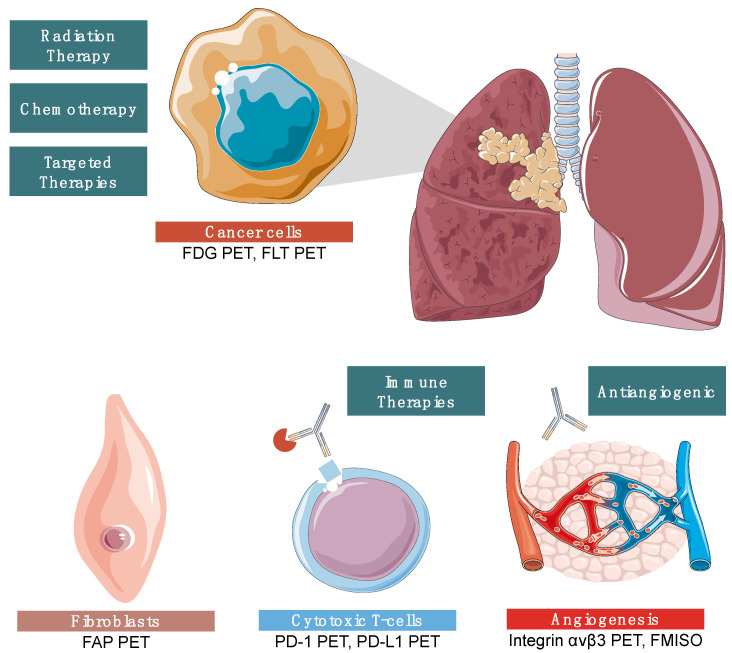
Overview of the available imaging and therapy options for patients with NSCLC. Treatment now includes chemotherapy, radiation therapy, targeted therapy, and immunotherapy. PET imaging and molecular radiotherapy targets include FDG, FLT, FAP, PD-1, PD-L1, integrins, and fMISO.

Novel therapies are changing the landscape of treatment for patients with NSCLC. Below, we detail some of the new and important targets that are improving the outcomes for patients. Figure 6 summarizes the important new imaging agents and therapeutic targets available for patients with NSCLC today.

## 4. Checkpoint Inhibitor Immunotherapy for Lung Cancer

Immune checkpoint inhibitors (ICI) (i.e., inhibitors of PD-1/PD-L1 and CTLA-4) are a class of immunotherapy that have been highly successful in treating lung cancer and have become an important part of the lung cancer treatment algorithm. 

### 4.1. Mechanism of Action

Effector T lymphocytes can attack and kill cancer cells; however, some cancer cells express PD-L1 on their surface as an immunosuppressive strategy, deactivating the T cell response upon the binding of their PD-L1 with the PD-1 receptors of T cells. PD-L1 is expressed in 35–95% of NSCLC patients and is associated with a poor prognosis [46,47]. Immune checkpoint inhibitors disrupt this interaction. Nivolumab was the first FDA-approved ICI, and since then, several other molecules have been developed and have shown therapeutic efficacy [48,49].

### 4.2. Clinical Trials and Implementation of ICIs as the New Standard of Care

Key monotherapy trials proved the efficacy of ICIs in lung cancer and established them as the new standard of care for certain patients. CheckMate017 (NCT01642004) and CheckMate057 (NCT01673867) were two phase III trials comparing docetaxel (DTX) and nivolumab as second-line treatments for squamous and non-squamous lung cancers. Both studies reported that nivolumab significantly increased the overall survival (OS) compared to DTX, leading to the approval of nivolumab as a second-line treatment of NSCLC [48,50]. On a pooled analysis, at 5-years post treatment the survival rate was above 10% with nivolumab, superior to all the available drugs for NSCLC at the time [49]. The IPSOS randomized phase III study showed that a monotherapy of anti-PD-L1 atezolizumab significantly improved patients’ median overall survival (OS) versus a single-agent chemotherapy establishing a new standard of care [51].

### 4.3. ICI Combinations and ICI + Chemoradiotherapy (CRT)

The KEYNOTE-189 trial reported a significantly higher progression-free survival (PFS) (i.e., 8.8 mo vs. 4.9 mo, and a HR 0.52) with platinum-based chemotherapy and pembrolizumab together versus chemotherapy alone in metastatic NSCLC [52], resulting in its approval as a first-line treatment for advanced-stage NSCLC. The CheckMate227 trial (NCT02477826) demonstrated that nivolumab plus ipilimumab as a first-line of treatment resulted in a greater overall survival than chemotherapy, leading to FDA approval of the combination as a first-line treatment for stage IV or recurrent NSCLC in 2020 [53]. The phase III PACIFIC trial (NCT02125461) demonstrated a robust overall survival benefit of continued PD-L1 inhibitor durvalumab administration, as a consolidation treatment after chemoradiation for patients with unresectable stage III NSCLC [54]. The PACIFIC2 trial (NCT03519971) is currently testing the efficacy of the combination of CRT and durvalumab, instead of sequential therapy. Additionally, the POSEIDON trial (NCT03164616) led to the FDA approval of anti CTLA-4 tremelimumab in combination with durvalumab and platinum-based chemotherapy for metastatic NSCLC in 2022 [55]. 

### 4.4. ICI and Targeted Therapy

Zhou et al. evaluated the combined effects of ICI and targeted therapy against VEGFR2 (i.e., apatinib and carrelizumab) and found the combination to have synergistic anticancer effects [56]. A combination therapy of the multiple tyrosine kinase inhibitor lenvatinib and pembrolizumab also demonstrated synergistic effects in NSCLC [57]. 

There is a strong rationale for the combination of ICIs with antiangiogenic treatment, as the VEGF/VEGFR pathway is involved in angiogenesis and is able to inhibit T cell effector function. The combination of the PD-1 inhibitor sintilimab and anlotinib demonstrated encouraging results as a first-line therapy in patients with advanced lung cancer [58]. 

### 4.5. Side Effects of ICIs

Most common side effects of ICIs include fatigue, itching, skin rash and diarrhea [59]. Anti-CTLA-4 treatment is more frequently associated with colitis and hypophysitis whereas pneumonitis and hypothyroiditis are primarily associated with anti-PD-1 treatment. Serious side effects can be treated with high doses of corticosteroids; however, if not addressed early they can be irreversible and fatal [60].

### 4.6. Challenges of ICIs

Despite promising results, there are still challenges and limitations with ICI treatments. The response rate to single agents is not as high as that of targeted therapies [61]. ICIs also appear less effective among patients harboring driver mutations or patients receiving steroid treatments [62]. The use of steroids prior to starting ICI treatment reduces their efficacy [63,64,65] and leads to a shorter survival [66]; however, the use of steroids as a treatment against IRAEs during ICI treatment was not reported to interfere with ICI efficacy [67,68].

There is still the need for a better understanding of the molecular mechanisms of resistance to ICIs and improved IRAE detection and management. New immune checkpoints are targeted beyond PD-1 and CTLA-4 [69]. For example, CITYSCAPE, a phase II trial with Tiragolumab + Atezolizumab (NCT03563716) for previously untreated, locally advanced, unresectable or metastatic NSCLC patients, reported promising results in 2022 [70]. A phase II trial combining the anti-LAG-3 agent, immutep, and pembrolizumab (NCT03625323) for first-line metastatic NSCLC patients unselected for PD-L1, also showed encouraging results [71].

## 5. EGFR Inhibitors

Approximately 15% of NSCLC tumors in patients from the United States bear an epidermal growth factor receptor (EGFR) mutation. EGFR mutations activate the tyrosine kinase (TK) domain of EGFR exon 19 or 20 [5], resulting in unregulated tumor growth.

### 5.1. First Generation EGFR Inhibitors

Targeted EGFR therapy plays an important role in cancer therapy for patients bearing this mutation and can prolong the patient survival time. The first generation of EGFR inhibitors, gefitinib and erlotinib, were approved by the FDA as the first-line treatment for EGFR-mutant NSCLC patients in 2015 and 2013, respectively. Mechanistically, gefitinib and erlotinib are reversible inhibitors of both mutated and wild-type EGFR [72,73]. The targeted first-line therapy of gefitinib or erlotinib can result in disease control for 11–14 months [72]; however, approximately 60% of patients develop a second EGFR mutation (frequently in the gatekeeper residue T790M) against which the first generation EGFR inhibitors are not effective [72]. 

### 5.2. Second Generation EGFR Inhibitors

Second-generation EGFR-TKIs, dacomitinib and afatinib, were developed in an effort to prevent mutation to a resistant clone and to delay the use of non-targeted therapies. They are irreversible inhibitors forming covalent bonds in the kinase domain of EGFR and other Her-family receptors [73]. The FDA approved afatinib in 2013 as a first-line therapy for non-resistant EGFR mutation del 19, L858R, and other rare mutations (e.g., S768I, L861Q and/or G719X). In 2018, dacomitinib was approved as a first-line therapy by the FDA for the treatment of NSCLC for patients with EGFR exon 19 deletion or exon 21 L858R. A phase III clinical trial (ARCHER 1050) in 2013–2015 compared the effectiveness of dacomitinib to the first generation gefitinib [74], and found dacomitinib significantly improved the progression-free survival over gefitinib in the first-line treatment of EGFR mutant NSCLC [74]. 

In 2021, the FDA granted accelerated approval to two drugs for patients with locally advanced or metastatic NSCLC carrying EGFRex20ins mutations, after progression on or after platinum-based chemotherapy. The first was amivantamab-vmjw, a bispecific antibody directed against both EGFR and MET receptors [75]. In a multicohort clinical trial (NCT02609776), 81 patients receiving amivantamab-vmjw had an ORR according to RECIST 1.1 of 40% with a median response duration of 11.1 months. The second, mobocertinib, was the first oral-targeted EGFR-TKI for patients with locally advanced or metastatic NSCLC with EGFRex20ins mutations [76]. In a study of mobocertinib, AP32788–15–101, (NCT02716116), the ORR in 114 patients whose disease had progressed on or after platinum-based chemotherapy was 28% with a median duration of response of 17.5 months.

### 5.3. Third Generation EGFR Inhibitors

Osimertinib is a third generation EGFR inhibitor that works by sensitizing and antagonizing EGFR T790M-containing mutant NSCLC cells. Ramalingam et al. [77] reported a phase III clinical trial of 556 patients with previously untreated advanced NSCLC with an EGFR mutation who received either osimertinib or other EGFR TK inhibitors (i.e., gefitinib or erlotinib). The overall survival was 38.6 months in the osimertinib group and 31.8 months in the comparator group [77].

## 6. ALK Inhibitors

Anaplastic lymphoma kinase (*ALK*) is a gene whose rearrangement results in a genetic alteration in the *ALK* gene leading to abnormal protein production and cellular signaling, and which acts as a driver mutation in 5% of NSCLC [78]. 

### 6.1. First Generation ALK Inhibitors

In 2011, the FDA approved crizotinib for the treatment of advanced ALK-positive NSCLC. Shaw et al. conducted a phase III, open-label trial comparing crizotinib and chemotherapy in patients with ALK-positive lung cancer [79]. Their results showed crizotinib as superior to standard chemotherapy with a median progression-free survival of 7.7 months in the crizotinib group compared to 3.0 months in the chemotherapy group [79]. Rapid resistance to crizotinib develops within 1–2 years after treatment and limits its clinical use. The main reason for the resistance is a mutation of the *ALK* tyrosine kinase domain, including L1196 M and C1156Y mutations, which occurs in a third of patients [80].

### 6.2. Second Generation ALK Inhibitors

Second generation ALK inhibitors, namely, ceritinib and alectinib, were developed to overcome crizotinib resistance. The randomized phase III clinical trials, ASCEND 4 and ASCEND 5, showed that ceritinib was more effective than first-line and second-line chemotherapy for NSCLC [81]. In 2017, the FDA approved ceritinib for first-line NSCLC treatment. Alectinib has the advantage over both crizotinib and ceritinib in that it can cross the blood–brain barrier and, therefore, can potentially treat brain metastatic disease.

### 6.3. Third Generation ALK Inhibitors

Lorlatinib is a third generation ALK inhibitor, characterized by its distinct macrocyclic structure which is designed to cross the blood–brain barrier and offer greater coverage against ALK inhibitor resistant mutations than second generation ALK inhibitors [82,83]. The CROWN trial was a large-scale phase III trial of patients with advanced, ALK-positive non-small-cell lung cancer comparing the efficacy of lorlatinib to crizotinib, which demonstrated a significantly-greater progression-free survival for patients treated with lorlatinib compared to those treated with crizotinib, with no significant increase in the rate of adverse events [84].

Similarly, brigatinib is another newer-generation ALK inhibitor, which is unique because it demonstrates efficacy in cell lines with EGFR mutations [85]. In 222 patients refractory to brigatinib, there was a median progression-free survival of 16.7 months [86,87].

## 7. MET Inhibitors

Another well-studied receptor tyrosine kinase is called MET, which has been an important therapeutic target in lung cancer [88]. The MET receptor is encoded by the *MET* oncogene, located on the long arm of human chromosome 7 (7q31). When mutated, multiple signal transduction pathways become activated, including the RAS-mitogen-activated protein kinase (MAPK) cascade, the PI3K-AKT pathway, and the Signal Transducer and Activator of Transcription (STAT) and NF-κB pathway, involved in cell proliferation, differentiation, cell motility, invasion and survival [89].

MET tyrosine kinase inhibitors have been studied as potential lung cancer therapeutic agents. Crizotinib is a potent inhibitor of MET tyrosine kinase as well as the ALK tyrosine kinase [90]. Crizotinib was found to be superior to the standard chemotherapy regimen in patients who had already received platinum-based chemotherapy [79]. The PROFILE 1014 trial demonstrated significantly prolonged progression-free survival (i.e., 10.9 months; 95% confidence interval [CI], 8.3 to 13.9 months) compared with 7.0 months in the chemotherapy group (95% CI, 6.8 to 8.2 months). Crizotinib treatment was also associated with a significantly-higher response rate and significantly-greater improvements in the patient-reported measures of physical functioning, key lung-cancer symptoms (e.g., cough, dyspnea, chest pain, and fatigue), and global quality of life [91].

Compared to the nonselective type 1A MET inhibitor crizotinib, tepotinib, savolitinib, and capmatinib are highly-selective MET inhibitors which have shown promising antitumor activity in both animal models and human studies [92,93]. A phase II study of tepotinib in patients with an exon 14 skipping mutation demonstrated a partial response in about half of the patients, similar to prior therapies [93]. The phase II Savannah trial evaluated the combined efficacy of savolitinib in EGFR-mutant, MET-driven, advanced NSCLC in patients who had been previously treated with osimertinib. The preliminary results from this trial demonstrated a 49% objective response rate to this regimen [94]. A similar trial of capmatinib in 364 patients with an MET Exon 14 skipping mutation in NSCLC demonstrated a 41% overall response rate. Together, the results of trials of these newer targeting agents demonstrate that they have the potential to improve the outcomes for patients with MET mutations; however, more work is needed to improve their efficacy and to identify combined regimens that result in a response in a greater number of patients.

## 8. Other Targeted Inhibitors in NSCLC

### 8.1. HER2 Targeting in NSCLC 

Another tyrosine kinase receptor found overexpressed on NSCLC cells is ErbB-2 protein, more commonly known as HER2 (human epidermal growth factor 2 receptor). Three HER2-activating mechanisms have been described in NSCLC: gene mutation (1–4% of cases), gene amplification (2–5%) and protein overexpression (2–30%) [95]. *HER2* amplification and overexpression have been associated with pleural invasion and *HER2* mutation has been associated with central nervous system (CNS) involvement [96].

Trastuzumab is a monoclonal immunoglobulin G1 humanized murine antibody that binds to the extracellular IV domain of the HER2 receptor and, therefore, blocks its dimerization; however, studies evaluating trastuzumab efficacy have not shown a significant benefit for patients with NSCLC who overexpress the HER2 protein [97]. Thus, drugs targeting the HER2 receptor with cytotoxic effects beyond receptor engagement have been introduced. Trastuzumab–emtansine is an antibody-drug conjugate which combines trastuzumab with the cytotoxic microtubule agent emtansine (DM1). Trastuzumab–emtansine enters HER2-positive cells through receptor-mediated endocytosis [98]. A phase II trial which administered a high dose of trastuzumab–emtansine led to a partial response in 44% of patients with HER2 mutant NSCLC [99]; however, larger randomized studies are needed to demonstrate the true potential of these promising agents.

Trastuzumab deruxtecan is an antibody-drug conjugate which is comprised of the humanized HER2 monoclonal antibody linked to a topoisomerase I inhibitor [100]. A multicenter, phase II, international trial evaluating the efficacy of trastuzumab deruxtecan found an objective response in 55% of patients, with an average duration of response of 9.3 months [101]. Further studies of this agent are needed to evaluate its potential in a larger population of patients.

### 8.2. RET Signaling in NSCLC 

RET is encoded by the *RET* gene which is located near the centromere of chromosome 10q [101]. *RET* rearrangements are found in about 1% of NSCLCs and are more commonly found in young, female, non-smoking patients with adenocarcinoma [102]. 

#### 8.2.1. Cabozantinib

Cabozantinib is a multikinase inhibitor with activity against RET, in addition to ROS1, MET, VEGFR2, AXL, TIE2, and KIT [102]. Cabozantinib has received FDA approval for the treatment of medullary thyroid cancer, renal cell carcinoma, and hepatocellular carcinoma. A phase II clinical trial (NCT01639508) looked at the activity of cabozantinib in patients with RET-rearranged lung adenocarcinoma. Twenty-eight percent of patients who received cabozantinib demonstrated a partial response, supporting that RET is an actionable mutation in lung cancers bearing this mutation [103]. Ongoing clinical trials are assessing the safety and efficacy of cabozantinib in RET fusion-positive NSCLC (NCT01639508 and NCT04131543).

#### 8.2.2. Vandetanib

Vandetanib is an oral receptor tyrosine kinase inhibitor that potently inhibits RET, EGFR, and vascular endothelial growth factor receptor tyrosine kinase activity [104]. A recent phase II multicenter clinical trial assessed the efficacy of vandetanib in patients with advanced RET-rearranged NSCLC. Out of 17 eligible patients, 9 achieved an objective response. At the data cutoff, the median progression-free survival was 4.7 months [105].

#### 8.2.3. Selpercatinib

Selpercatinib is a highly-selective small molecular RET inhibitor that was designed to penetrate the central nervous system and has been tested as a treatment for NSCLC. This is particularly advantageous given the propensity for NSCLC to metastasize to the brain [106]. A clinical trial of selpercatinib in 105 patients with NSCLC found that in patients previously treated with platinum-based chemotherapy, 64% had an objective response, while in patients who were not previously treated, 85% had an objective response. In patients with intracranial metastatic disease, 91% exhibited an intracranial objective response [106]. These results demonstrate the promise of selpercatinib, although further work is needed to assess the efficacy and safety of this agent on a larger scale.

### 8.3. ROS1 targeting in NSCLC

Entrectinib received accelerated approval from the FDA in 2019 for adults with met-astatic NSCLC with ROS1-positive tumors and those with solid tumors that had a neurotrophic tyrosine receptor kinase (NTRK) gene fusion. Both are oncogenic drivers of various cancers, including NSCLC. Up to 36% of patients that are ROS1 fusion-positive have brain metastases at the time of diagnosis [107]. Efficacy in ROS1-positive metastatic NSCLC was investigated in 51 adult patients who received entrectinib. The ORR was 78% (95% CI: 65, 89) and response duration was 12 months or longer for 55% of patients.

## 9. Emerging Therapies

### 9.1. Bispecific Antibodies

While monoclonal antibodies are usually specific to one epitope, bispecific antibodies (bsAbs) are intended to bind two epitopes [108]. Epitopes may be found on a tumor itself or on immune effector cells [108,109]. By engaging two different sites, bsAbs better target a specific tumor [109], thereby decreasing the potential side effects [110]. Additionally, having two different binding sites means that two different functional pathways for a given tumor can be targeted, thereby reducing the risk of treatment resistance [111]. Pairing epitopes potentiates a functionality that cannot be accomplished by using a combination of monoclonal antibodies, including immune cell-redirecting activity [75,112]. The phase I CHRYSALIS trial examined the bispecific antibody, amivantamab, for advanced NSCLC with *EGFR* exon20 insertion mutation, and demonstrated a 40% response rate and a progression-free survival of 8.3 months, resulting in a breakthrough therapy designation [75]. 

### 9.2. CAR T Cell Therapy

Chimeric antigen receptor (CAR) T cells are a type of immunotherapy utilizing T cells modified to recognize and bind to cancer cell antigens [113]. Given the upregulated expression of EGFR in NSCLC tissue, EGFR is a highly-promising target in CAR T cell development. Biopsies have demonstrated that CAR T cells targeting EGFR can infiltrate tumor tissue and promote EGFR-targeted cytotoxicity [113]. A phase I trial using EGFR-targeting CAR T cells showed that 7 out of 11 patients demonstrated either a partial response or stable disease. Ongoing research will assess the role of CAR T cell therapy as a combination therapy for lung cancer. Additional targets are also currently being investigated such as mesothelin, mucin, and CEA, which may enhance efficacy via the targeting of multiple pathways to reduce the likelihood of acquiring resistance. Multiple target CARs and combination treatments such as low-dose radiation therapy may help to overcome some of the limitations of CAR T cell therapy currently being encountered in clinical trials against solid tumors [114].

### 9.3. Molecular Targeted Radiotherapy

Molecular targeted radiotherapy directs radioactive particles to cancer cells to deliver a focused radioactive payload that can arrest tumor growth by conjugating therapeutic radionuclides to tumor restricted markers. Studies have shown that they also activate the immune system and can be combined with immunotherapy agents to provide a more robust response [115,116]. One popular molecular target in the development of this therapy has been FAP, expressed on cancer-associated fibroblasts, but not in normal tissues. Preclinical data has demonstrated FAP targeted radiotherapy to be a promising strategy for lung cancer, melanoma, and pancreatic cancer [36,117].

### 9.4. Alternate Drug Delivery Systems

Due to the dose-limiting side effects of many agents, alternate drug delivery systems are being developed to target lung cancer therapies. Molecular targeted radiotherapy, as detailed above, conjugates a radiotherapeutic ligand to a targeting molecule, thereby delivering a focused radioactive payload to a tumor [118,119]. Other agents, such as AVA600, use the same type of targeting strategies to localize a pro form of drug to tumors, then use a cell’s own machinery to convert the pro-drug to its active form [120]. Specific to lung cancer, nanoparticles offer an alternative promising option given the large surface area of the lungs, and that particles will be absorbed in first-pass metabolism [121]. Numerous types of nanoparticles have been studied, such as extracellular vesicles, micelles, liposomes, and carbon nanotubules. These agents have the potential to provide alternate lung cancer treatments with high specificity and limited side effects.

### 9.5. Inhalational Therapy

The main routes for administering therapeutic agents for lung cancer are orally or intravenously. Novel agents that can be delivered directly to the lungs via inhalation has been a subject of interest recently. Such therapies would require lower doses and exhibit fewer systemic toxic effects; however, inhalation agents would also present unique problems, namely, their complex pharmacological properties and toxicity from lung retention [122]. 

Several existing agents, such as doxorubicin and cisplatin, have been utilized in clinical trials, but the side effects on the lungs were too severe to pursue further trials [123]. Agents such as amodiaquine have been repurposed, and show anti-tumor effects when loaded into nanoparticle polymers for inhaled treatment versus controls [124]. With the surge in novel delivery methods, there is reason for optimism with delivering effective agents to primary lung cancers and lung metastases of other types of cancer in novel ways [125]. 

## 10. Current State of NSCLC Diagnosis and Treatment

The treatment for early-stage, locally advanced, and metastatic NSCLC has evolved beyond chemotherapy with the incorporation of immunotherapies and targeted treatments. In patients with operable early-stage NSCLC, surgery remains the primary treatment modality for cure. Adjuvant treatment is recommended based upon the pathologic stage and findings at the time of surgery [126]. Patients with locally advanced, inoperable stage III NSCLC are typically treated with definitive concurrent chemoradiation followed by adjuvant durvalumab for up to 12 months. 

As above, numerous new types of therapy have become available in the past 10 years, and many are continuing to be developed as evidenced by numerous ongoing clinical trials (Table 2). Checkpoint inhibitors are now a major pillar in lung cancer therapy. In patients with specific driver mutations, drugs targeting those proteins have become the mainstay of therapy. Additional new agents, currently in clinical trials and in the preclinical stages of development, will likely further transform this field and offer options to patients for whom none of the available therapies are effective. Molecular testing has now become the norm and is important in guiding patient management. After 4–6 cycles of initial therapy, patients with stable or responsive disease transition to maintenance therapy with chemotherapy and/or immunotherapy. Targeted radiotherapies may fill the clinical niche for patients in whom other therapies are non-effective. Because many therapies target tumor-specific markers that are present in all tumors, such as FAP, these agents promise to be useful in a broader patient population than any of the presently available targeted therapies.

**Table 2:** Current clinical trials for each drug class.

Drug Class	Clinical Trials	Description
ICI	Ramucirumab and Atezolizumab After Progression on Any Immune Checkpoint Blocker in NSCLC (RamAtezo-1) [127]	Assessing for a therapeutic advantage to administering ICI and VEGEF inhibitor to patients previously receiving immunotherapy.
EGFR Inhibitors	An Open-label, Single-Arm, Phase II Trial of Stereotactic Body Radiotherapy for Patients With Residual Oligometastases of NSCLC After 3rd Generation EGFR-TKIs [128]	After administering 3rd generation EGFR-TKIs, SBRT will be given until regression or intolerance.
ALK Inhibitors	Activity of Lorlatinib Based on ALK Resistance Mutations on Blood in ALK Positive NSCLC Patients Previously Treated With 2nd Generation ALK Inhibitor [129]	Identifying alterations in the ALK gene that may confer benefit from ALK inhibitor therapy.
MET Inhibitors	Assessment of Anti-tumor and Safety in Glumetinib in Patients With c-MET-positive Non-Small Cell Lung Cancer [130]	Local or metastatic NSCLC patients with a METex14 mutation, not undergoing chemotherapy.
RET Inhibitors	A Phase I Study of Oral LOXO-260 in Patients With RET Fusion-Positive Solid Tumors, Medullary Thyroid Cancer, and other Tumors with RET Activation Refractory to Selective RET Inhibitors [131]	Testing of a next-generation RET inhibitor, which has exhibited *in vitro* effect against RET-positive tumors with acquired resistance mutations.
Bispecific Antibodies	AK112 Neoadjuvant/Adjuvant Treatment for Resectable NSCLC [132] AK104 Monotherapy as Neoadjuvant and Adjuvant Therapy for Resectable Non-small Cell Lung Cancer [133]	Investigating the effect of an anti-PD1 and VEGF bispecific antibody in combination with platin and paclitaxel chemotherapy. AK104 is a tetravalent bispecific antibody targeting PD-1 and CTLA-4; this study aims to evaluate its efficacy and safety profile.
CAR T Cells	Study of CXCR5 Modified EGFR Targeted CAR-T Cells for Advanced NSCLC [134]	Early Phase I study investigating the pharmacokinetics and anti-tumor effect of a novel CAR T cell.

## 11. Conclusions

Collectively, advances in imaging, molecular testing, and therapy options have improved the outcomes for patients with lung cancer. According to a new study, NSCLC mortality rates have fallen sharply in the US in recent years [135]. As patient outcomes improve, the future directions of lung cancer treatment include understanding how and why resistance to treatment develops and identifying other actionable molecular targets.
